# *Escherichia coli* Heat-Labile Detoxified Enterotoxin Modulates Dendritic Cell Function and Attenuates Allergic Airway Inflammation

**DOI:** 10.1371/journal.pone.0090293

**Published:** 2014-03-17

**Authors:** I-Ping Lin, Yu-Shen Hsu, Ssu-Wei Kang, Miao-His Hsieh, Jiu-Yao Wang

**Affiliations:** 1 Institute of Microbiology & Immunology, College of Medical, National Cheng-Kung University, Tainan, Taiwan; 2 Development Center for Biotechnology, Taipei, Taiwan; 3 Department of Pediatrics, College of Medicine, National Cheng Kung University, Tainan, Taiwan; 4 Graduate Institute of Integrated Medicine, School of Chinese Medicine, China Medical University, Taichung, Taiwan; University of Rochester Medical Center, United States of America

## Abstract

Various mutant forms of *Escherichia coli* heat-labile enterotoxin (LT) have been used as a mucosal adjuvant for vaccines, as it enhances immune responses to specific antigens including antigen-specific IgA antibodies when administrated intranasally or orally. We hypothesized that a detoxified mutant form of LT, LTS61K, could modulate dendritic cell (DC) function and alleviate allergen-induced airway inflammation. Two protocols, preventative and therapeutic, were used to evaluate the effects of LTS61K in a *Dermatophagoides pteronyssinus* (Der p)-sensitized and challenged murine model of asthma. LTS61K or Der p-primed bone marrow-derived dendritic cells (BMDCs) were also adoptively transferred into Der p-sensitized and challenged mice. Intranasal inoculations with LTS61K or LTS61K/Der p decreased allergen-induced airway inflammation and alleviated systemic T_H_2-type immune responses. Bronchoalveolar lavage fluid (BALF) and sera from LTS61K/Der p-treated mice also had higher concentrations of Der p-specific immunoglobulin (Ig) A than those of other groups. In vitro, BMDCs stimulated with Der p underwent cellular maturation and secreted proinflammatory cytokines interleukin (IL)-6 and tumor necrosis factor (TNF)α In contrast, Der p-stimulated BMDCs that were pretreated with LTS61K showed decreased IL-6 and TNFα production and were less mature. Intratracheal adoptive transfer of LTS61K- or LTS61K/Der p-primed BMDCs into Der p-sensitized mice reduced inflammatory cell infiltration and T_H_2-type chemokines in BALF and alleviated airway inflammation in treated mice. LTS61K influenced DC maturation and decreased inflammatory cytokine production. Moreover, LTS61K/Der p induced increased Der p-specific IgA production to decrease allergic T_H_2 cytokine responses and alleviated airway inflammation in Der p-sensitized mice. These results suggest that the immunomodulatory effects of LTS61K may have clinical applications for allergy and asthma treatment.

## Introduction

Allergic asthma is a chronic airway inflammatory disease that is characterized by eosinophil infiltration, bronchial epithelium damage, and airway hyper-reactivity (AHR), which result from immunopathogenic T_H_2-type responses to environmental allergens, such as house dust mites (HDMs) [1]. Hypersensitivity to HDM (*Dermatophagoides sp.*) allergens is one of the most common allergic responses and affects up to 85% of asthmatics [2]. Sensitization to indoor allergens is the strongest independent risk factor associated with asthma. Additionally, >50% of children and adolescents with asthma are sensitized to HDMs. Although allergen-specific CD4^+^ T_H_2 cells orchestrate the HDM allergic response through the induction of immunoglobulin (Ig)E directed against mite allergens, activation of innate immune responses, such as those of dendritic cells (DCs) in the airway mucosa, also plays a critical role in HDM-induced allergic inflammation [3].

DCs are highly specialized antigen-presenting cells that regulate innate and adaptive immune responses, thus playing an important role in the pathogenesis of asthma and allergic rhinitis [4], [5]. DCs can distinguish among different pathogenic compounds through their expression of various pattern-recognition receptors that recognize specific pathogen-associated molecular patterns. Indeed, an ultimate adaptive T-cell immune response may be determined by the differentiation and maturation status, expression profile of costimulatory molecules, and specific pattern-recognition receptors that are used for the recognition of antigens by DCs [6]. This role of DCs for determining a particular immune response may provide a target for alleviating or possibly modulating airway inflammation in allergic asthma patients.

Because of its high immunogenicity, heat-labile enterotoxin (LT), which is secreted by *Escherichia coli*, has been developed and modified for clinical use as a mucosal vaccine adjuvant [7]. Detoxified and mutant forms of LT used as a mucosal adjuvant induce antigen-specific mucosal IgA secretion, which can protect experimental animals from pathogen infections and various adverse immune responses [8]. For example, LTS63 can induce T_H_1 immune responses against influenza virus, whereas LTR72 can protect mice from *Bordetella pertussis* by activating T_H_2 immune responses [8]–[10].The mechanisms underlying these different types of immune responses caused by various mutant forms of LT remain unclear. However, it is quite possible that this may due to different degrees of interaction between DCs and mutant LT, resulting in different immune responses.

In this study, we used a mucosal immunomodulator, LTS61K (United States Patent No.: US 8, 110, 197 B2). The 61 position of the A subunit was mutated from Ser to Lys. However, this mutation does not affect it stability and binding affinity to its receptor, GM1. This newly developed, detoxified LT enterotoxin has been used as an adjuvant for the nasal influenza vaccine (Phase I study, Institutional Review Board code: 201112125MSA, National Taiwan University Hospital, R.O.C). In this study, we investigated the effects of LTS61K in an allergic asthma murine model and its involvement in the maturation and function of DCs. Our results showed that intranasal administration of LTS61K or LTS61K in combination with HDM allergen, decreased AHR and attenuated the cardinal features of allergen-induced airway inflammation. In addition, LTS61K/HDM also induced allergen-specific IgA production. These effects of LTS61K may have resulted from modulation of DC function to reverse allergic immune responses, as shown by our in vivo and in vitro results. Thus, a detoxified mutant form of LT, LTS61K, may have clinical applications for allergy and asthma treatment as an immunomodulator.

## Materials and Methods

### Ethics Statement

This animal study was granted an Affidavit of Approval of Animal Use Protocol by National Cheng Kung University (IACUC No.: 1021390). Mice were kept in specific-pathogen free conditions and provided a standard diet and water at the animal facilities of the National Cheng-Kung University Laboratory Animal Center. Mice were intraperitoneally injected with an overdose of Zoletil 50 (Vibrac, Carros, France) plus Rompun at sacrifice.

### Animals and reagents

Female BALB/c mice (aged 6–8 weeks) were obtained from the National Cheng-Kung University Laboratory Animal Center. *Dermatophagoides pteronyssinus* (Der p) extract (1 g of lyophilized whole body extract in diethyl ether; Allergon, Engelholm, Sweden) was dissolved in pyrogen-free isotonic saline, filtered with a 0.22-µm filter, and stored at −80°C before use. The lipopolysaccharide (LPS) concentration of prepared Der p was <0.96 EU/mg (limulus amebocyte lysate test, E-Toxate; Sigma-Aldrich, St. Louis, MO, USA). LTS61K was produced by the Development Center for Biotechnology (New Taipei City, Taiwan) following cGMP procedures, dissolved in non-LPS-containing PBS, and stored at 4°C before use. The LPS concentration of LTS61K was <0.918 EU/mg, and the binding affinity to GM1 (Kd) was 10^−9^–10^−11^.

### *In vivo* experimental protocol (Figure S1)

Der p extract was mixed with incomplete Freund's adjuvant (Sigma-Aldrich) at equal volumes and injected intradermally at 40 µg Der p per mouse on days 0 and 7. Mice were intratracheally (i.t.) injected with 50 µg Der p under light anesthesia with Zoletil 50 plus Rompun on day 14 and examined on day 17 as in a previous study [11]. For the therapeutic protocol, after airway inflammation was induced, mice were divided into different groups and intranasally (i.n.) treated with either normal saline (10 µL per mouse), LTS61K (10 µg per mouse), or LTS61K (10 µg) mixed with Der p (20 µg) per mouse under light anesthesia thrice within 1 week. After the last i.n. treatment, mice were i.t. treated again with 50 µg Der p on day 21 and examined 3 days later (**Fig. S1A**). For the preventative protocol, mice were divided into the groups described above and i.n. treated once per week for 3 weeks. After 2 weeks, mice were sensitized, airway inflammation was induced by Der p as described above, and were examined on day 45 (**Fig. S1B**).

### Generation, treatment, and analysis of bone marrow-derived dendritic cells

Bone marrow cells were flushed from the femurs and tibias of naïve mice or Der p-induced allergic asthma mice and cultured in RPMI 1640 medium supplemented with 10% heat-inactivated fetal bovine serum (FBS), 50 U/mL penicillin, 50 µg/mL streptomycin, and 20 ng/mL murine recombinant granulocyte-macrophage colony-stimulating factor (GM-CSF; Pepro Tech) at 1–2×10^6^ cells/10 mL for 8 days [12]. Non-attached cells were harvested, their concentration was adjusted to 1×10^6^ cells/mL, and then cultured with Der p, LPS, or LTS61K for 24 hours. For pretreatment experiments, cells were treated with LTS61K for 4 hours, washed with phosphate-buffered saline (PBS), and then treated with LPS or Der p.

### Adoptive transfer of bone marrow-derived dendritic cells

An adoptive transfer protocol was used and modified as described elsewhere [13]. Briefly, BMDCs were cultured with Der p (20 µg), LTS61K (10 µg), or LTS61K (10 µg) mixed with Der p (20 µg) for 24 hours. Then, each mouse was i.t. treated with 5×10^5^ DCs on day 0. Treatment with 20 µg Der p/mouse was i.n. administered on day 1 to day 5, after which each mouse was i.t. challenged with 50 µg Der p on day 6. AHR was assessed on day 8 and mice were sacrificed on day 9 (**Fig. S1C**).

### Measurement of airway resistance

Invasive analysis of lung function was performed for mice anesthetized with ketamine hydrochloride (0.08 µg/g; Sigma-Aldrich) and xylazine hydrochloride (0.008 µg/g; Sigma-Aldrich) in 0.9% NaCl. A steel cannula was inserted into the trachea, after which the mouse was placed in a chamber to measure lung resistance (R_L_) with exposure to increasing doses of methacholine using a FinePointe system (Buxco Electronics, Inc., Wilmington, NC, USA). AHR was assessed by noninvasive measurement of dynamic airway resistance (Penh value) with increasing aerosol concentrations of acetyl-β-methylcholine chloride (methacholine; Sigma-Aldrich) using unrestrained whole body plethysmography (Buxco).

### Bronchoalveolar lavage fluid sampling

Mice were sacrificed by intraperitoneal injection of an overdose of Zoletil 50 plus Rompun. BALF was sampled with two 1-mL aliquots of saline. A total of 1.8–1.9 mL BALF was collected consistently. Total BALF cells were stained with trypan blue and counted under a microscope.

### Der p-specific IgG1, IgG2a, and IgA determinations

Whole blood from each mouse was collected and centrifuged at 2500 rpm for 20 minutes. Serum was collected and stored at −80°C before analysis. Enzyme-linked immunosorbent assay (ELISA) plate wells were coated with Der p (10 µg) in coating buffer (15 mM Na_2_CO_3_ and 35 mM NaHCO_3_, pH 9.6) at 4°C overnight, washed with 0.05% Tween 20 in PBS (PBST), and blocked with 0.5% bovine serum albumin (BSA; Sigma-Aldrich). Sera or BALF samples were diluted to appropriate concentrations and added to wells at 4°C overnight. After washing with PBST, goat anti-mouse IgA conjugated horseradish peroxidase (HRP; 1∶10000, Novus Biologicals), goat anti-mouse IgG1 conjugated HRP, or goat anti-mouse IgG2a or conjugated HRP (IgG1, 1∶10000, IgG2a, 1∶5000, Bethyl) was added. Finally, color was developed with TMB (tetramethylbenzidine) substrate (Clinical Science Products).

### Cytokine measurements

The concentrations of IL-12, TARC (CCL17), TNF-α, and eotaxin-1 (CCL11) in BALF and IL-6 and TNFα in the supernatants of cultured BMDCs were determined using ELISA kits (IL-12, TNFα, eotaxin, TARC, and IL-6, R&D DuoSet).

### Lung histochemistry

The lungs of each mouse were first inflated with 1 mL formalin (Sigma-Aldrich) via the trachea, then excised and fixed in 4% buffered formalin. Lung tissue was embedded in paraffin, cut into 5-mm sections, and stained with hematoxylin and eosin. Inflammatory cell infiltration and lung architecture were assessed by light microscopy. The mucus secretion level was detected by periodic acid-Schiff (PAS; Sigma-Aldrich) staining. Lung sections were deparaffinized and hydrated in water, and stained with periodic acid for 5 minutes. After rinsing with distilled H_2_O, lung sections were stained with Schiff reagent for 15 minutes and washed in tap water. Finally, the sections were counterstained in Mayer's hematoxylin, dehydrated, and mucus secretion was assessed by light microscopy.

### Flow cytometric analysis

After treatment for 24 hours, BMDCs were harvested and stained with fluorescein FITC-conjugated anti-mouse CD11c, PE-conjugated anti-mouse CD11b, PE-conjugated anti-mouse CD80, PECy5-conjugated anti-mouse CD86, and PECy5-conjugated anti-mouse major histocompatibility (MHC) class II antibodies (eBioscience). The FITC Annexin V apoptosis detection kit (BD Pharmingen) was used for cell death studies. Cells were washed with PBS and fixed with 1% paraformaldehyde (Merck). Fluorescently labeled cells were analyzed by flow cytometry (FACScan, BD).

### Statistical analysis

Results are given as the mean ± standard error of the mean (SEM). Between-group comparisons for total cells, cytokines, Der p-specific IgA in BALF, Der p-specific antibodies in serum, the change of R2 group cell numbers and cells surface marker expression level by LTS61K, and Der p and LPS stimulation of BMDCs from naïve and Der p-sensitized mice were made using one-way analysis of variance (ANOVA) followed by Bonferroni multiple comparison tests. Student's t-tests were used for comparisons of IL-6 and TNF-α levels, cell numbers, and cell surface marker expression after different treatments in naïve mice. The Mann-Whitney U test was used to compare Der p-specific IgA levels in BALF after the adoptive transfer of BMDCs. Penh values and R_L_ were compared between groups by two-way ANOVA followed by Bonferroni multiple comparison tests. A p-value of <0.05 was considered statistically significant.

## Results

### Intranasal treatment with LTS61K or LTS61K/Der p after allergen challenge alleviates pathological T_H_2-type immune responses in sensitized mice

To evaluate the therapeutic potential of LTS61K in our allergen-induced asthma model, Der p-sensitized mice were i.n. treated with either LTS61K or LTS61K mixed with Der p (LTS61K/Der p) thrice in 1 week after airway challenge with Der p allergen (**Fig. S1A**). The control group contained Der p-sensitized mice treated with vehicle (saline) using the same protocol as that used for treatment groups.

**Figure 1:**
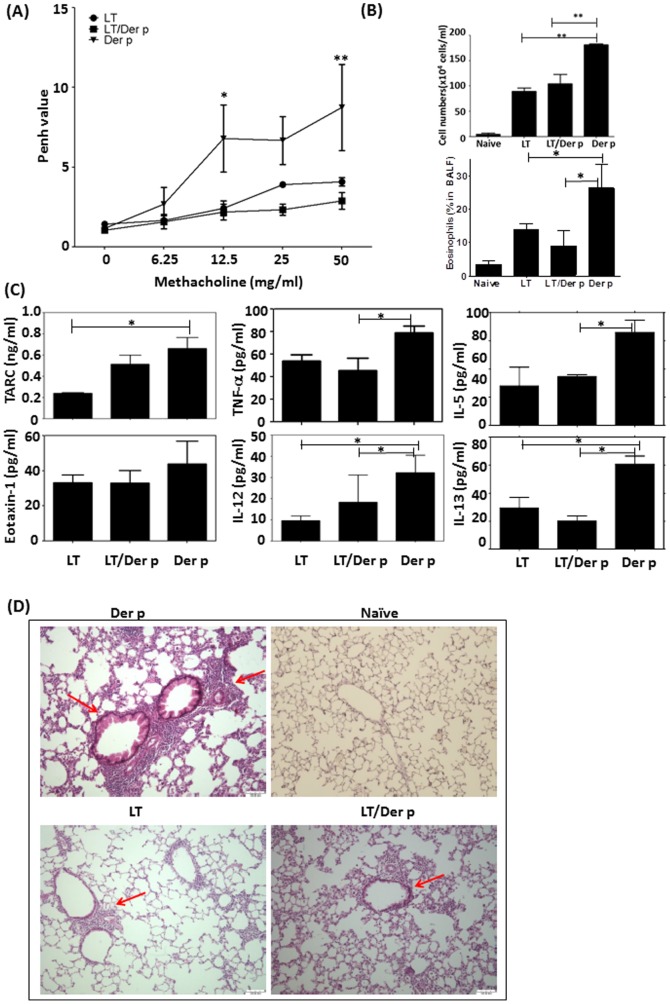
Intranasal treatment with LTS61K or LTS61K/*Dermatophagoides pteronyssinus* (Der p) alleviates Der p-induced allergic asthma in mice. Mice were sensitized to and boosted with Der p mixed with incomplete adjuvant. After an initial airway challenge, intranasal treatments were given thrice in 1 week. After a second airway challenge, mice were sacrificed 3 days later and assessed for airway resistance based on (**A**) Penh values, (**B**) total infiltrated cells, and (**C**) cytokines in bronchoalveolar lavage fluid (BALF). (**D**) Hematoxylin and eosin staining. Red arrows indicate inflammatory cell infiltrates in the parenchyma and damaged epithelial cells in the bronchi. Results represent the mean ± standard error of the mean for n = 6–12 mice per group. *p<0.05, **p<0.01. Data are shown for 3 representative experiments.

LTS61K and LTS61K/Der p treatment groups had decreased airway resistance compared to control mice as assessed by methacholine challenge (**Fig. 1A**). In BALF from LTS61K- and LTS61K/Der p-treated mice, the total number of infiltrating cells (**Fig. 1B**), and percentages of eosinophils were decreased significantly as well as TNFα and Th2-type cytokines, particularly TARC (CCL17), IL-5, and IL-13 (**Fig. 1C**), as compared to BALF from non-treated sensitized mice. Interestingly, LTS61K or LTS61K/Der p treatment resulted in decreased IL-12 levels in BALF as compared to controls (**Fig. 1C**). Lung histology results also showed significantly reduced peribronchiolar inflammatory cell infiltration and goblet cell hyperplasia and improvement in bronchial epithelial linings in LTS61K and LTS61K/Der p treatment groups as compared to the non-treated group (**Fig. 1D**, red arrow).

**Figure 2:**
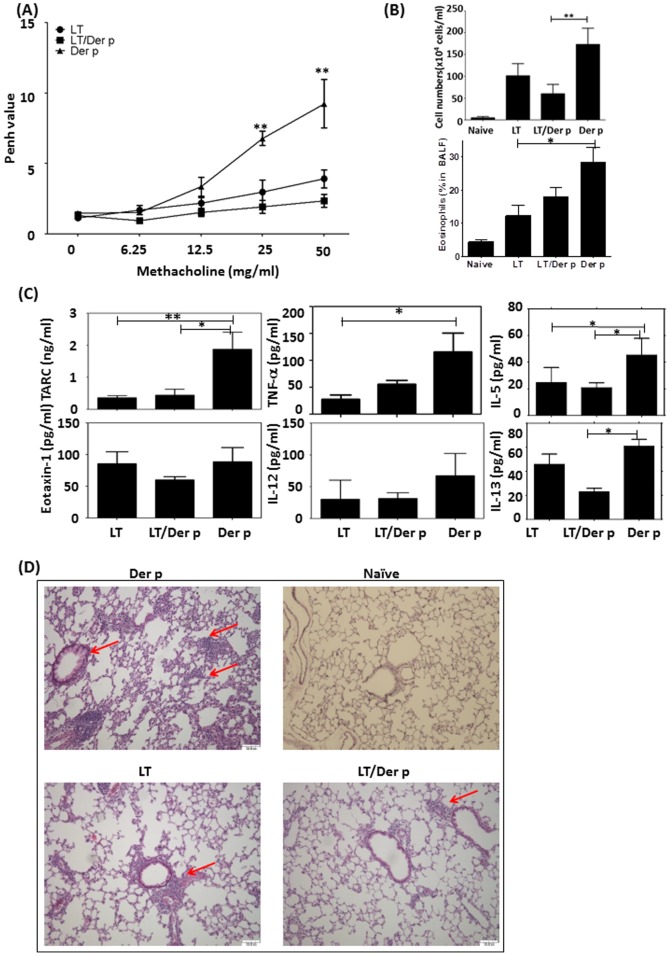
Intranasal pretreatment with LTS61K or LTS61K/*Dermatophagoides pteronyssinus* (Der p) alleviates Der p-induced allergic asthma in mice. Mice were intranasally treated once in 1 week. After 2 weeks, mice were sensitized to and boosted with Der p mixed with incomplete adjuvant. After a final airway challenge, mice were sacrificed 3 days later and assessed for airway resistance based on (**A**) Penh values, (**B**) total infiltrated cells, and (**C**) cytokines in bronchoalveolar lavage fluid (BALF). (**D**) Hematoxylin and eosin staining. Red arrows indicate inflammatory cell infiltrates in the parenchyma and damaged epithelial cells in the bronchi. Results represent the mean ± standard error of the mean for n = 6–12 mice per group. *p<0.05, **p<0.01. Data are shown for 3 representative experiments.

To evaluate the preventative effect of LTS61K in our Der p allergen-induced asthma model, LTS61K or LTS61K/Der p was first administered i.n. once per week for 3 weeks. Then, 2 weeks later, Der p allergen sensitization and challenges were initiated (**Fig. S1B**). Both LTS61K- and LTS61K/Der p-treated mice showed reduced methacholine-induced AHR compared to non-treated Der p sensitized and challenged mice (**Fig. 2A**). There was also significantly decreased total inflammatory cell infiltration (**Fig. 2B**) and percentage of eosinophils in the LTS61K/Der p-treated mice relative to non-treated control mice. In BALF from LTS61K- and LTS61K/Der p-treated mice, there were significantly decreased IL-5, IL-13, and TARC (CCL17) chemokine levels as compared to non-treated mice (**Fig. 2C**). Lung histology results showed significantly reduced peribronchial infiltrates and improved bronchial epithelia in LTS61K/Der p-treated mice compared to non-treated controls (**Fig. 2D**, red arrow). In addition, i.n. treatment with LTS61K alone, and an increased frequency of the airway challenge protocol from 3 to 6 times within 2 weeks, also reduced lung resistance and mucus secretion (red arrow, which points to the purple site of the airway, indicates the mucus secretion by goblet cells) in allergic asthma mice (**Fig. S2**).

### LTS61K combined with Der p intranasal administration induces antigen-specific IgA systemic and local production

**Figure 3:**
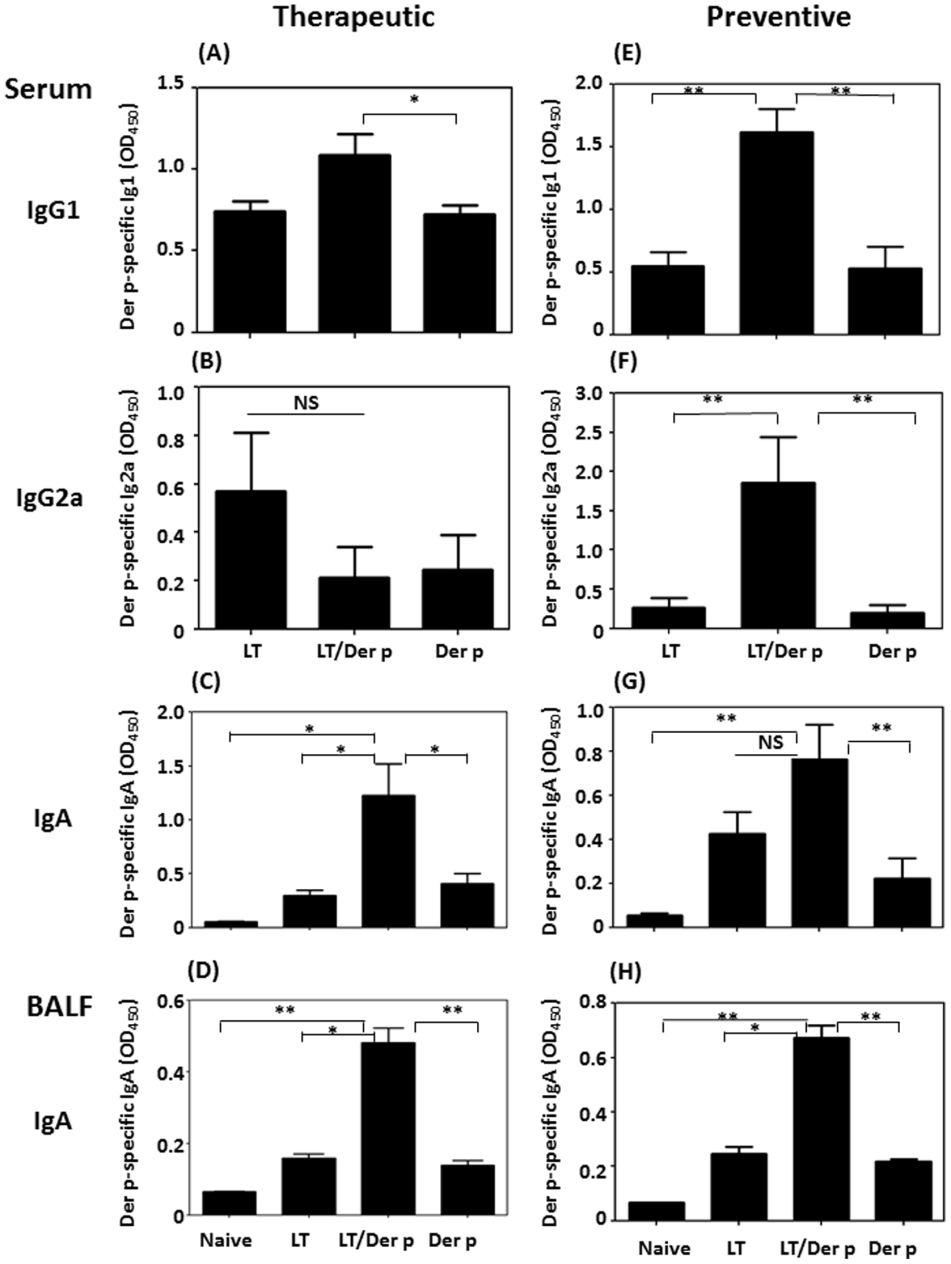
*Dermatophagoides pteronyssinus* (Der p)-specific antibody profiles among different animal protocols. (**A–D**) Therapeutic protocol. (**E–H**) Preventative protocol. (**A**) and (**E**) Serum IgG1. (**B**) and (**F**) Serum IgG2a. (**C**) and (**G**) Serum IgA. (**D**) and (**H**) Bronchoalveolar lavage fluid (BALF) IgA. Results represent the mean ± standard error of the mean for n = 6–12 mice per group. *p<0.05, **p<0.01. NS, not significant.

Next, we examined the different antibody isotypes produced in allergen-induced mice. Der p-specific IgG1 levels in the LTS61K/Der p treatment group were higher than those in the other groups (**Fig. 3A**). In contrast, Der p-specific IgG2a levels did not differ between the groups (**Fig. 3B**). There were no statistically changes of total IgE and Der p-specific IgE in sera between LTS61K-treaed and non-treated allergic mice of both preventive and therapeutic protocols (**Fig. S3**). Local allergen-specific IgA production may influence the interactions between allergens and immune cells in the airway and thus alleviate airway inflammation in allergen-induced allergic inflammation mice. Interestingly, intranasal treatment with LTS61K/Der p increased the Der p-specific IgA levels both in BALF and sera (**Fig. 3C, 3D**). In the preventative protocol using LTS61K or LTS61K/Der p treatment, there were also significantly increased levels of Der p-specific IgG1 (**Fig. 3E**), IgG2a (**Fig. 3F**), and IgA in LTS61K/Der p-treated mice compared to those of non-treated control mice (P<0.01) (**Fig. 3G, 3H**).

### LTS61K affects dendritic cell maturation and cytokine production

**Figure 4:**
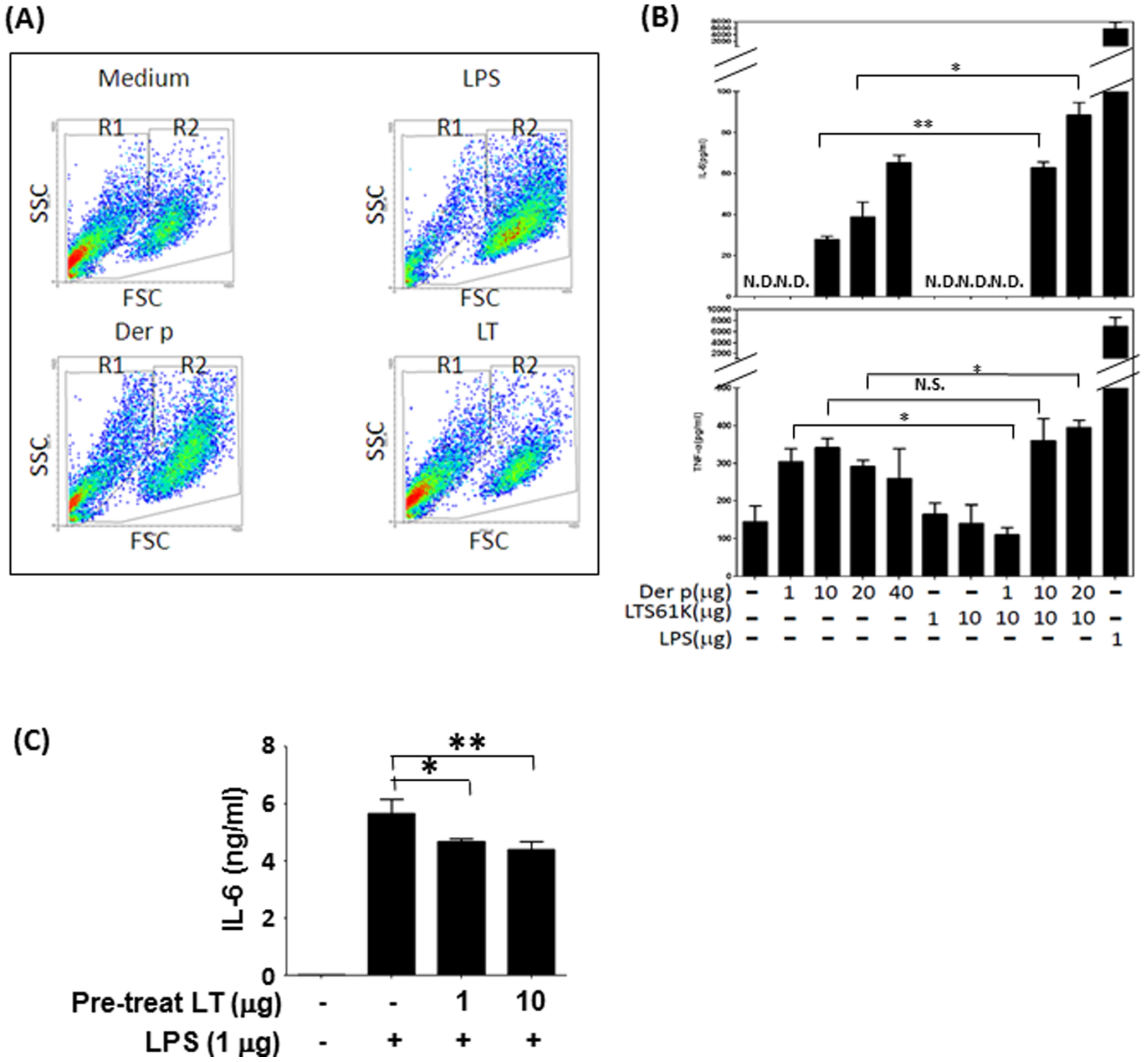
LTS61K affects bone marrow-derived dendritic cell (BMDC) maturation. BMDCs from BALB/c naïve mice were cultured with either *Dermatophagoides pteronyssinus* (Der p) (20 µg), LTS61K (10 µg), or lipopolysaccharide (LPS; 1 µg) for 24 hours, and then analyzed by flow cytometry. (**A**) Cell population distributions. Results for 1 representative experiment of 3 are shown. (**B**) Interleukin (IL)-6 and tumor necrosis factor (TNF)α levels. (**C**) IL-6 secretion by BMDCs was determined after pretreatment with 1 µg or 10 µg LTS61K for 4 hours and 1 µg LPS for 24 hours. N.D., not determined. NS, non-significant, Results represent the mean ± standard error of the mean for n = 6–8 mice per group. *p<0.05, **p<0.01 (Student's t-test).

DCs are the most potent antigen-presenting cells and are linked with innate and adaptive immunity. Based on cellular volumes and light scattering properties of particles, we found that GM-CSF-generated BMDCs from naïve mice could be divided into2 groups (i.e., R1 and R2) by flow cytometry (**Fig. 4A**). Upon further analysis, the R2 cell group exhibited higher expression of CD80 and CD86 and intermediate MHC class II expression compared to the R1 group of cells (**Fig. S4A** and Table 1. In addition, both R1 and R2 cells expressed CD11b, with R2 cells showing higher expression of CD11c (**Fig. S4B**). Table 1 shows the percentages of these 2 groups of cells and the surface marker expression of BMDCs under different treatment conditions.

**Table 1:** Cell numbers (%) and cell surface marker expression levels (MFI) with different treatments and subgroups.

Treatment	Subgroup	Cell Numbers Percentage			MFI		
		Total	CD11b	CD11c	CD80	CD86	MHC class II
**Medium**	R1	60.1±2.1%	83.5±3.1%	10.1±3.1%	22.1±2.6	23.4±1.7	76.3±13.7
	R2	39.8±2.1%	95.4±3.2%	63.2±4.1%	50.7±6.9	61.1±7.0	392.9±189.2
**LPS**	R1	26.8±1.5%	86.8±4.5%	12.8±4.5%	25.3±1.1	27.9±1.0	69.2±3.0
	R2	72.9±1.5%	92.9±1.5%	84.9±4.5%	#102.7±30.0	#170.1±46.1	1246±404.7
**Der p**	R1	46.7±1.2%	86.7±4.2%	16.7±3.2%	20.5±1.3	23.8±1.1	81.6±12.2
	R2	53.2±1.1%	93.2±3.1%	72.3±3.1%	71.2±16.0	#106.9±13.0	1274±412.5
**LTS61K**	R1	61.9±0.8%	91.9±1.5%	11.9±1.1%	21.6±2.5	25.2±3.4	69.4±17.4
	R2	38.0±0.8%	88.0±1.5%	40.5±1.1%	54.6±8.9	69.0±7.8	485.7±139.9

# p<0.05 compared to medium-only groups (Student's t-test). Der p, *Dermatophagoides pteronyssinus*; LPS, lipopolysaccharide.

When BMDCs were treated with LPS or Der p for 24 hours, there were increasing shifting of cells from R1 to R2 fractions as well as with statistically significant increase of cells expressing CD80 and CD86 as compared to those in medium-only group. Although MFI of MHC Class II were also increased in LPS- or Der p-treated BMDCs, but not to a statistically difference (Table 1). In contrast, LTS61K-teated BMDCs did not have significantly increase in the expression of CD80, CD86, and MHC Class II and with nearly the same percentage of cell populations in R1 and R2 as in medium-only group (Table 1). Interestingly, LTS61K treatment did not induce cell death as assayed by Annexin V expression in flow cytometry. (**Fig. S5**). Thus, these data suggest that Der p and LPS, but not LTS61K, induce DC maturation as shown in the shifts from R1 to R2 and increased expression of co-stimulatory molecules in treated BMDCs.

Proinflammatory cytokine (IL-6 and TNFα) production by Der p- and LPS-stimulated BMDCs was higher than that by LTS61K-stimulated BMDCs (**Fig. 4B**). However, treating BMDCs with both LTS61K and Der p may have increased the production of these proinflammatory cytokines by BMDCs. In contrast, BMDCs that were pretreated with LTS61K significantly inhibited LPS (1 µg)-induced IL-6 cytokine production compared to non-pretreated BMDCs (**Fig. 4C**). We have also measured IL-10 and IL-12 production from BMDCs after stimulation with Der p and LPS. We found there were no significant changes of IL-6 and IL-12 levels with or without pretreatment of LTS61K (data not shown). Thus, LTS61K may inhibit LPS-induced BMDC maturation and proinflammatory cytokine production.

### Pretreatment with LTS61K decreases MHC class II expression on BMDCs isolated from allergen-sensitized and challenged mice

**Figure 5:**
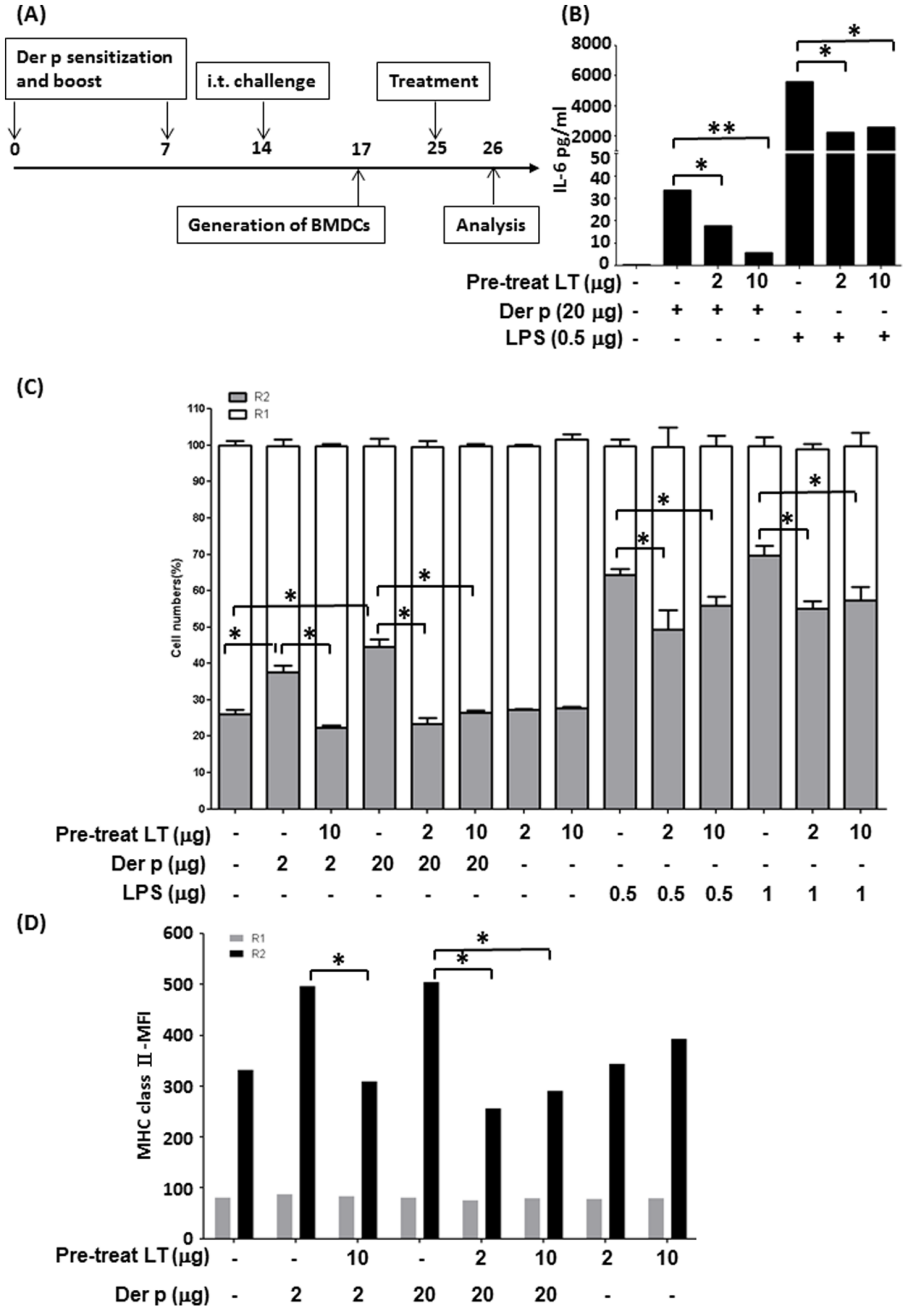
Pretreatment with LTS61K inhibits maturation of *Dermatophagoides pteronyssinus* (Der p)-induced bone marrow-derived dendritic cells (BMDCs) from Der p-sensitized mice. (**A**) Ex vivo model. Der p-induced allergic asthma mice were sacrificed 3 days after the last challenge. Bone marrow cells were harvested and cultured with 20 ng/mL granulocyte-macrophage colony-stimulating factor (GM-CSF) for 8 days. BMDCs were harvested, pretreated with LTS61K (2 or 10 µg) for 4 hours, and then cultured with Der p (2 or 20 µg) and lipopolysaccharide (LPS; 0.5 or 1 µg) for 24 hours. (**B**) Interleukin (IL)-6 levels. (**C**) R1 and R2 cell numbers. (**D**) Major histocompatibility (MHC) class II expression in R1 and R2 cells. Results represent the mean ± standard error of the mean for n = 6–8 mice per group. *p<0.05, **p<0.01, #, non-significant. Results for 1 representative experiment of 3 are shown.

Next, we generated BMDCs from allergen-sensitized and challenged asthma model mice as described previously (**Fig. 5A**). FcεR1 expression on BMDCs from sensitized mice was higher than that from naïve mice, which indicated that these mice were in an “atopic” state (data not shown). After Der p allergen challenge, significantly increased numbers of mature BMDCs (R2 group) were isolated from Der p-sensitized mice.

In an in vitro study of BMDCs isolated from Der p-sensitized and challenged mice, pretreatment with LTS61K (2 µg and 10 µg) for 4 hours not only inhibited Der p- (20 µg) or LPS (0.5 µg)-induced IL-6 cytokine production (**Fig. 5B**), but also decreased the cell numbers and Der p- or LPS-induced BMDC maturation in the R2 group (**Fig. 5C**) and inhibited R2 cell MHC class II expression (**Fig. 5D**).

### Adoptively transferred LTS61K-pulsed BMDCs alleviate airway inflammation

**Figure 6:**
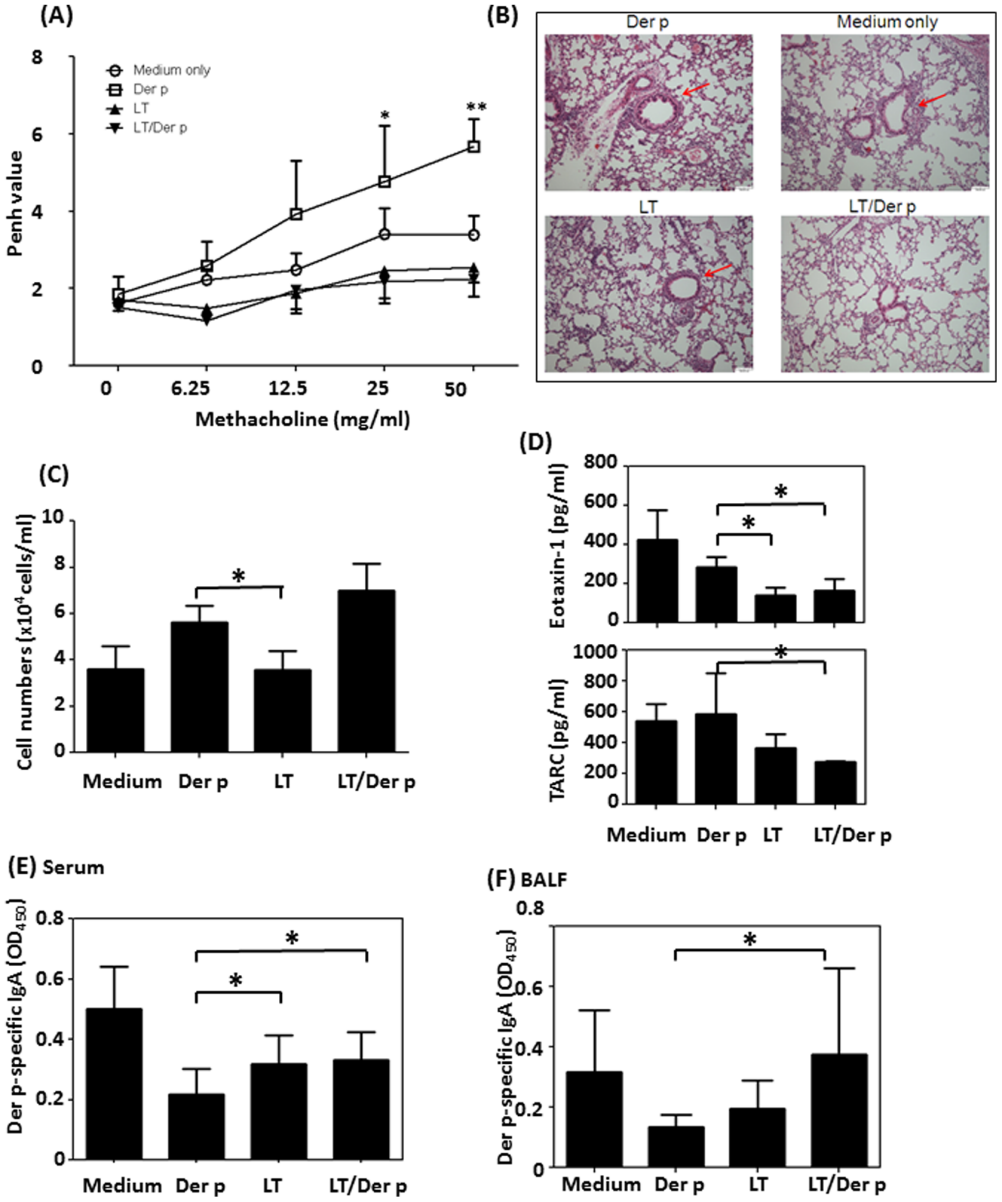
Adoptive transfer of LTS61K- and LTS61K/*Dermatophagoides pteronyssinus* (Der p)-pulsed bone marrow-derived dendritic cells (BMDCs) alleviates Der p-induced allergic asthma in mice. BMDCs from naïve mice were treated with either Der p, LTS61K, or Der p mixed with LTS61K for 24 hours. Mice were intratracheally administered 5×10^5^ BMDCs/mouse, intranasally administered 20 µg Der p for 5 days, and then intratracheally administered 50 µg Der p on day 6. Penh values were determined on day 8, and mice were sacrificed on day 9. (**A**) Penh values. (**B**) Hematoxylin and eosin staining. Red arrows indicate inflammatory cell infiltrates in the parenchyma and damaged epithelial cells in the bronchi. (**C**) Total infiltrated cells. (**D**) Cytokines in bronchoalveolar lavage fluid (BALF). Der p-specific IgA in (**E**) serum and (**F**) BALF. Results represent the mean ± standard error of the mean for n = 6–8 mice per group. *p<0.05, **p<0.01. Results for 1 representative experiment of 3 are shown.

Although the mechanisms by which DCs initiate T_H_2 responses are not completely understood, previous studies showed that DCs may be potent antigen-presenting cells that initiate allergic asthma [14]. To test the hypothesis that LTS61K could induce different maturation states and functions of DCs that influence the immunologic responses to allergens, we adoptively transferred LTS61K- or LTS61K/Der p-primed BMDCs into naïve mice and challenged these mice with Der p allergen (**Fig. S1C**). Significantly decreased methacholine-induced airway resistance was observed (**Fig. 6A**) and peribronchiolar inflammation was alleviated to a greater extent (**Fig. 6B**, red arrow) in Der p-challenged mice with adoptively transferred LTS61K- or LTS61K/Der p-primed BMDCs compared to those that received Der p-primed BMDCs. There were no significant differences in total cell numbers between these groups of mice (**Fig. 6C**). T_H_2 chemokine (eotaxin 1 and CCL11) levels of mice with adoptively transferred LTS61K-primed BMDCs were lower than those of mice that received Der p-primed BMDCs (**Fig. 6D**). In addition, Der p-specific IgA levels in sera (**Fig. 6E**) and in BALF (**Fig. 6F**) differed between these groups of mice adoptively transferred with different primed-BMDCs.

## Discussion

In this study, the effects of LTS61K, a detoxified enterotoxin, was evaluated in an allergen-induced murine asthma model. Our results showed that mice that were i.n. treated with LTS61K combined with Der p had increased Der p-specific IgA levels either locally (BALF) or in the systemic circulation (sera). In addition, LTS61K strongly suppressed the cardinal features of allergic airway inflammation when applied locally to the upper airway mucosa (i.n. administration) through a mechanism involving DC function and IgA-secreting protective B cells. Interestingly, despite allergen-specific IgA production, i.n. treatment with LTS61K alone also alleviated AHR and airway inflammation, as with LTS61K/Der p treatment.

Although we cannot exclude an indirect stimulatory effect of LTS61K due to its possible interactions with other cell types such as macrophages or bronchial epithelial cells, we believe that LTS61K promulgated these effects by altering DC function and maturation, thereby programming DCs to induce IgA-producing B cells and regulatory T cells. This conclusion is based on the following results. In vivo adoptive transfer of LTS61K/Der p-pulsed DCs induced Der p-specific IgA production. In addition, in vitro culture of BMDCs pretreated with LTS61K and exposed to Der p resulted in a less mature state and decreased inflammatory cytokine production compared to Der p stimulation alone. To evaluate the effects of these mutant detoxified enterotoxin on the maturation and functions of BMDCs, LPS has been frequently used as positive control to evaluate the response of BMDCs. In addition, the positive control of LPS also denoted the effect of Der p on BMDCs was through the biological activity of Der p itself, and rule out the possibility of contamination of tiny LPS in Der p extracts.

As shown in Fig. S3, we could not detect statistically significant changes of total IgE and allergen (Der p)-specific IgE in sera between LTS61K-treated and non-treated allergic mice of both preventive and therapeutic protocols. It is presumed that the anti-allergic effect of LTS61K is not on the immune-regulatory change of Th1 and Th2 immune responses, but may on the immunological tolerance effect that, although in the sensitized (high IgE level) state, but still improve the pathological and physiological event of allergen-induced airway inflammation in LT-treated allergic mice. Several studies have shown that non-allergic individuals exhibit some immune tolerance responses to allergens, as occasionally reflected by high serum levels of allergen-specific IgA and IgG4 in the absence of specific IgE [15], [16]. It was also previously shown that IgA administered directly into the lungs could capture and neutralize inhaled allergens [17]. Recently, Smits et al. [13] showed that protective IgA-producing B cells could be induced by locally administrated cholera toxin (CT) B into the bronchial mucosa and protected against allergic inflammation in OVA-sensitized and challenged mice. Several studies have found that LT and its mutant forms (e.g., LTK63 and LTR72) might induce antigen-specific mucosal IgA secretion that protects experimental animals from pathogenic infections [8], [9], [10].

CT and LT are AB5 enterotoxins that are produced by *Vibrio cholerae* and enteropathic *E. coli* and are the primary causative agents of cholera and traveler's diarrhea, respectively [18]. Owing to their high immunogenicity, CT and LT have been prepared and modified as powerful mucosal adjuvants, although their cellular targets and mechanisms of action are unknown. Indeed, administration of LT by virtually any mucosal route will first activate innate immune responses, including the secretion of inflammatory cytokines, DC recruitment, and DC activation and initiation of their antigen-presenting function [19]–[23].

Emerging evidence suggests that DCs are one of the principal cell types that mediate the adjuvant effects of these toxins in vivo. Bagley et al. investigated the effects of CT and LT on the maturation of human monocyte-derived DCs (MDDCs) in vitro. They found that CT and LT predominantly inhibited IL-12 and TNF-α production by MDDCs in the presence of LPS, and that an enzymatically active A domain was necessary for both CT and LT to induce MDDC maturation that was strictly cyclic adenosine monophosphate dependent [24]. CT has been noted to have adjuvant activity and induce T_H_2-type immunity in the mucosa [25]–[27]. Krishnamoorthy et al. reported that both HDMs and CT elicited T_H_2 and T_H_17 immune responses through activation of the c-Kit signal transduction pathway in DCs [28]. In addition, CT and LT are highly homologous (80% amino acid homology) and share the same receptor [18], which indicates that LT and HDMs may have some interactions in vitro.

Therefore, several methods have been used to reduce or eliminate LT toxicity while retaining its immunogenicity and DC modulation effects. A recent study discovered that LTB, the B subunit of LT, protected mice from OVA-induced allergic asthma by inducing regulatory T cells [29]. In this study, our detoxified LTS61K contained a mutated subunit A, which included an amino acid substitution at position 61 (LTS61K), did not have any effect in the inducing regulatory T cells in the lungs of LTS61K or LTS61K/Der p treated allergic mice (Fig. S6), which suggested that anti-allergic effect of LTS61K was not directly through increasing regulatory T cell numbers in the inflammatory lungs.

Our in vitro BMDCs stimulation study showed that LTS61K decreased the cell numbers and the expression of costimulatory molecules, CD80, CD86, and MHC class II, and reduced the percentages of CD11c^+^ mature DCs in Der p-sensitized mice (Table 1). In the analysis of cultured bone-marrow derived DCs (BMDCs) has clearly shown two populations, R1 and R2, according to their size and granulations. There is significant higher of MHC class II expression of R2 cells than R1 cells after LPS or Der p stimulation (Fig. 5D). Fig. S4A also shows the MFI changes of MHC class II expression between Der p and LTS61K/Der p are majority on the R2 cell population. We suspected that only R2 represent functional DCs that with strong expression of MHC Class II and CD80/CD86 molecular that revealed in Table 1 and Fig. 5C and 5D. Fig. S4B and Table 1 shows the data of CD11b and CD11c staining for the two different subsets of BMDCs from naïve mice stimulated with LPS, Der p, and LPS. We found both R1 and R2 expressed CD11b and CD11c, the majority of CD11c expressions were in R2 cells, but to a lesser degree in R1 cells. The origin or characteristic of R1 cells are presently unknown. Whether R1 cells belonged to DCs category needs further studies to confirm. In the Fig. S5, we have also shown that R1 cells are not dead cells, for the expression annexin V did not increased after LTS61K or LPS treatment.

A previous study showed that CD11c^+^ DCs, which are derived from bone marrow, were the main cells that initiated T_H_2-type allergic asthma [14]. Thus, LTS61K may act on BMDCs through down-regulation of MHC class II expression, influence the adaptive immune functions associated with DCs, and alleviate allergen-induced airway inflammation. Moreover, mice that received adoptively transferred LTS61K-primed BMDCs also exhibited reduced airway inflammation and T_H_2 chemokine levels in BALF compared to those that received adoptively transferred Der p-primed BMDCs.

Our previous studies showed that Der p allergen could directly activate innate immune cells, including alveolar macrophages [30] and mast cells [31], and could induce T_H_2 cytokine responses without prior in vitro or in vivo sensitization. Other recent studies have suggested that allergen-induced DC activation and inflammation may involve toll-like receptors (TLRs) and/or C-type lectin receptors (CLRs) on antigen-presenting cells [32]–[35]. We have also shown that Der p allergen could directly activate human MDDCs via binding with and internalization of DC-SIGN, a CLR on DCs, and that after these DCs matured, they promoted the T_H_2 polarization of naive CD4^+^ T cells [36], [37].

Therefore, in addition to its high immunogenicity and mucosal adjuvant effects that induce antigen-specific adaptive immune responses, our findings that LTS61K had an immunoregulatory effect on mucosal immune responses to Der p allergen have broad implications for its preventative and therapeutic effects on allergic diseases. Further studies of LTS61K effects in allergic disease patients, particularly in conjunction with allergen-specific immunotherapy, are warranted for this new therapeutic modality.

## Supporting Information

Figure S1 Animal study models. (**A**) Therapeutic protocol. (**B**) Preventive protocol. (**C**) Bone marrow-derived dendritic cell (BMDC) adoptive transfer model.
(TIF)

Figure S2 Intranasal treatment with LTS61K alone inhibits airway hyperresponsiveness and mucus secretion in allergen-induced allergic asthma mice. (**A**) and (**D**) Animal model. Mice were sensitized to and boosted with *Dermatophagoides pteronyssinus* (Der p) as previously described. Intranasal treatments with LTS61K were followed by (**A**) 3 airway challenges or (**D**) 6 challenges in 2 weeks. After the last challenge, airway hyperresponsiveness was assessed by invasive measurement of dynamic airway resistance (Fine Pointe RC System, Buxco). (**B**) and (**E**). Lung tissues were collected and (**C**) and (**F**) periodic acid-Schiff (PAS) stained (red arrow, which points to the purple site of the airway, indicated mucus secretion by goblet cells) Results represent the mean ± standard error of the mean for n = 6–8 mice per group. **p<0.01. Results for 1 representative experiment of 3 are shown.
(TIF)

Figure S3 Total IgE and Dermatophagoides pteronyssinus (Der p)-specific IgE antibodies in the preventive and therapeutic animal protocols.
(TIF)

Figure S4 Bone marrow-derived dendritic cells from naïve mice were collected, harvested for 8 days, and stained for (**A**) CD80, CD86, and MHC class II and (**B**) CD11b and CD11c. n = 6 mice. Results for 1 representative experiment of 4 are shown.
(TIF)

Figure S5 Annexin V-PI staining. Bone marrow-derived dendritic cells from naïve mice were collected, harvested for 8 days, and treated with (A) medium only, (B) LTS61K, (C) *Dermatophagoides pteronyssinus* (Der p), and (D) lipopolysaccharide (LPS) for 24 hours. Cells were collected and stained with Annexin V-FITC and PI. n = 6 mice. Results for 1 representative experiment of 3 are shown.
(TIF)

Figure S6 (A) Flow cytometry analysis of CD4^+^/CD25^+^ cells in collagenase-digested lungs. (B). Percentages of CD4^+^CD25^+^ cells in the lungs of naïve and treated allergic mice.
(TIF)
