# Supplementary figures and images for: Escherichia coli Heat-Labile Detoxified Enterotoxin Modulates Dendritic Cell Function and Attenuates Allergic Airway Inflammation
Source: PLoS One. 2014 Mar 17;9(3):e90293. doi: 10.1371/journal.pone.0090293 (PMC3956462; doi:10.1371/journal.pone.0090293)

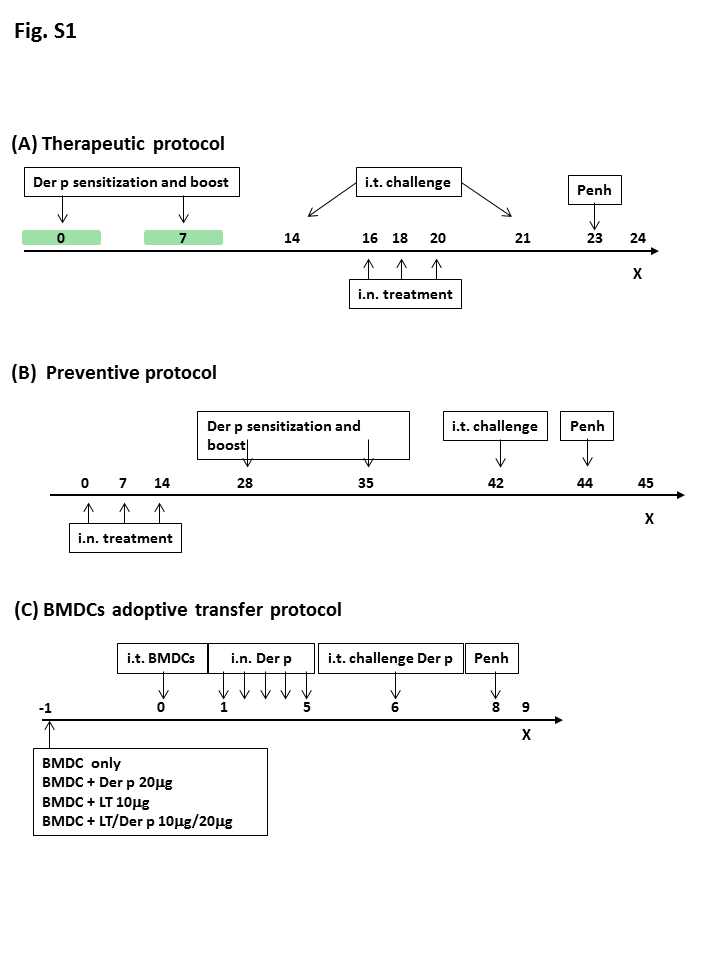

Supplement: Figure S1 — Animal study models. (A) Therapeutic protocol. (B) Preventive protocol. (C) Bone marrow-derived dendritic cell (BMDC) adoptive transfer model. (TIF) [file pone.0090293.s001.tif]

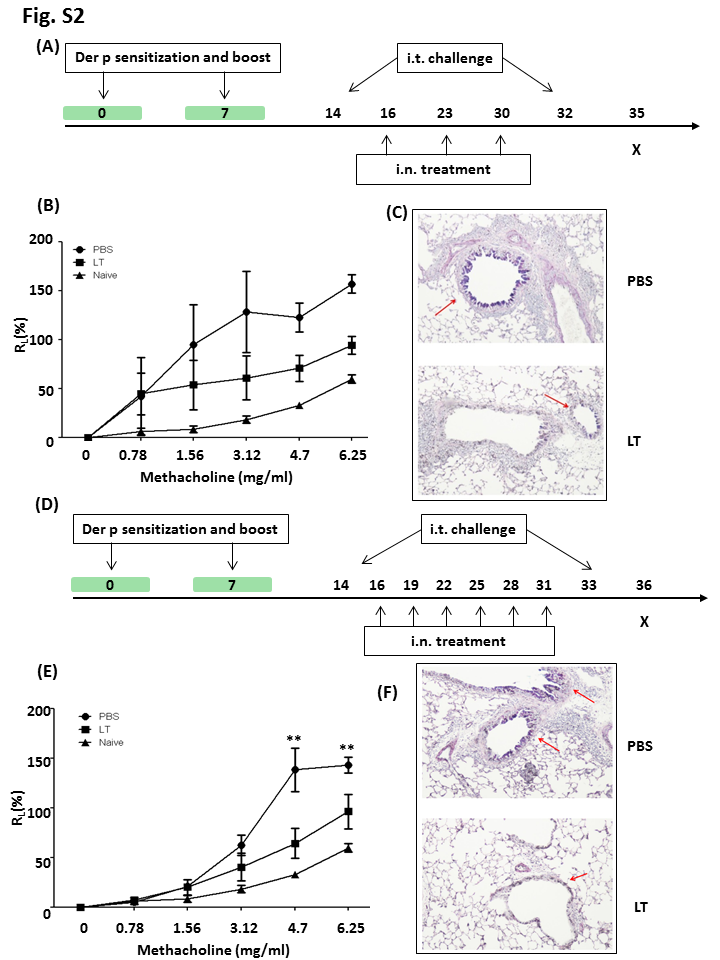

Supplement: Figure S2 — Intranasal treatment with LTS61K alone inhibits airway hyperresponsiveness and mucus secretion in allergen-induced allergic asthma mice. (A) and (D) Animal model. Mice were sensitized to and boosted with Dermatophagoides pteronyssinus (Der p) as previously described. Intranasal treatments with LTS61K were followed by (A) 3 airway challenges or (D) 6 challenges in 2 weeks. After the last challenge, airway hyperresponsiveness was assessed by invasive measurement of dynamic airway resistance (Fine Pointe RC System, Buxco). (B) and (E). Lung tissues were collected and (C) and (F) periodic acid-Schiff (PAS) stained (red arrow, which points to the purple site of the airway, indicated mucus secretion by goblet cells) Results represent the mean ± standard error of the mean for n = 6–8 mice per group. **p<0.01. Results for 1 representative experiment of 3 are shown. (TIF) [file pone.0090293.s002.tif]

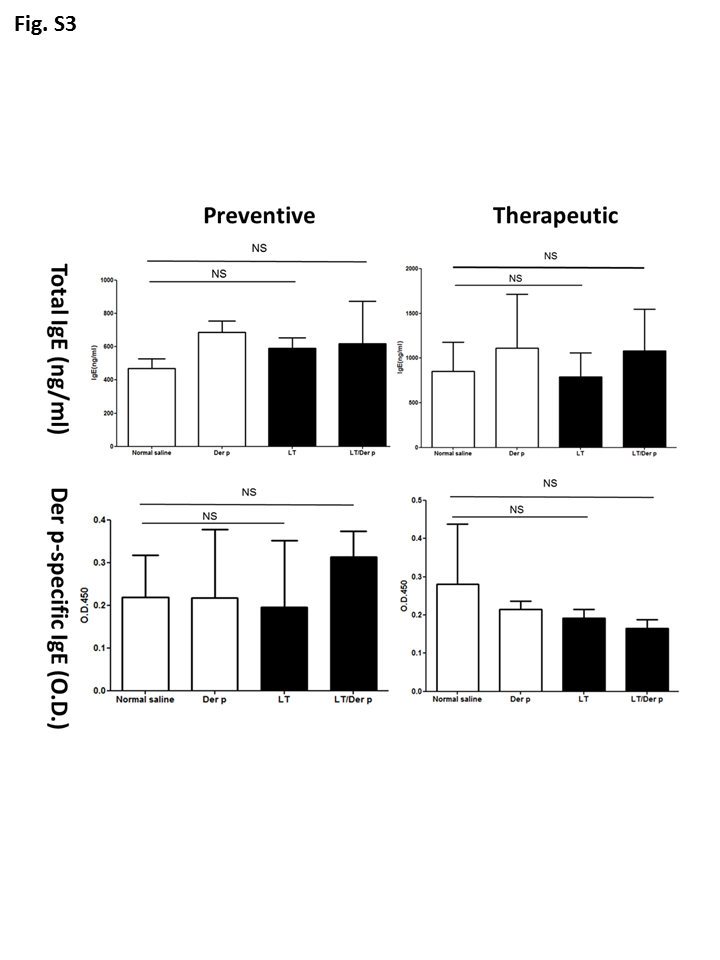

Supplement: Figure S3 — Total IgE and Dermatophagoides pteronyssinus (Der p)-specific IgE antibodies in the preventive and therapeutic animal protocols. (TIF) [file pone.0090293.s003.tif]

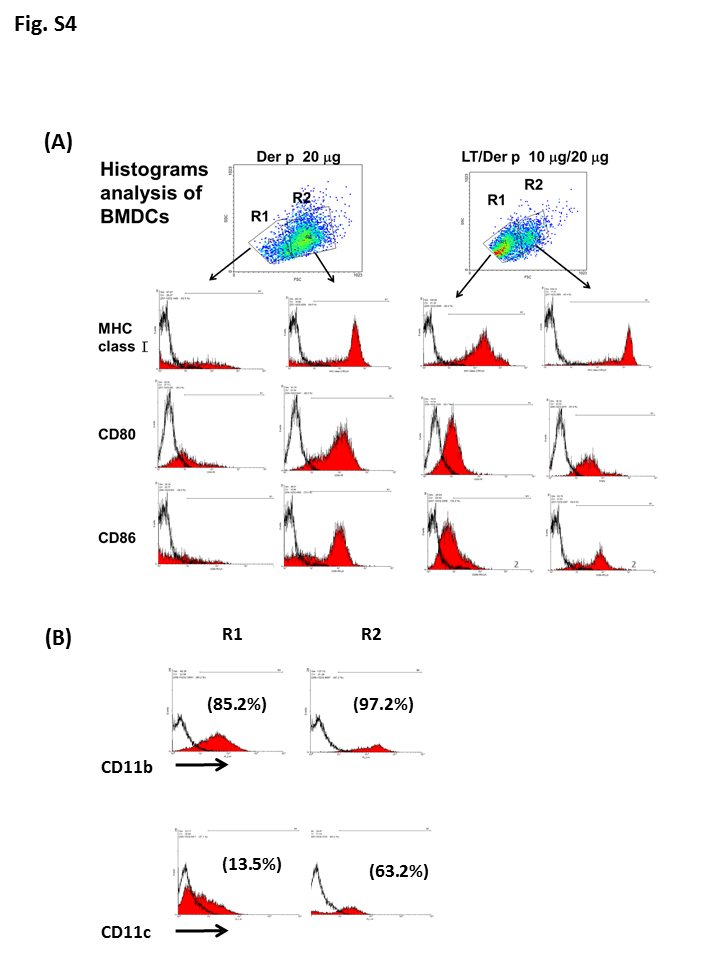

Supplement: Figure S4 — Bone marrow-derived dendritic cells from naïve mice were collected, harvested for 8 days, and stained for (A) CD80, CD86, and MHC class II and (B) CD11b and CD11c. n = 6 mice. Results for 1 representative experiment of 4 are shown. (TIF) [file pone.0090293.s004.tif]

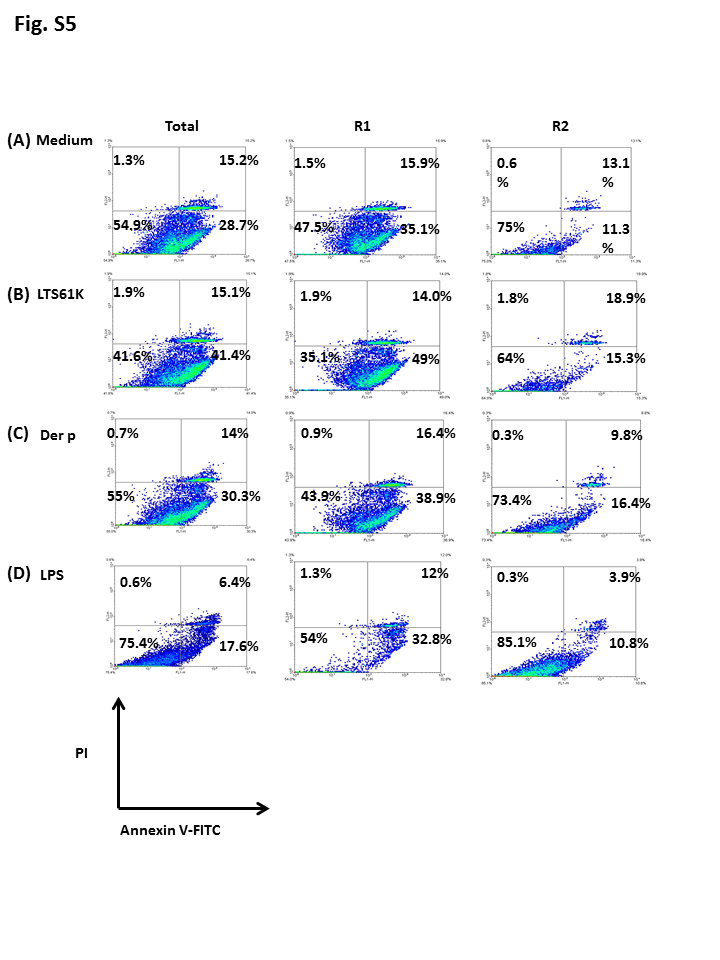

Supplement: Figure S5 — Annexin V-PI staining. Bone marrow-derived dendritic cells from naïve mice were collected, harvested for 8 days, and treated with (A) medium only, (B) LTS61K, (C) Dermatophagoides pteronyssinus (Der p), and (D) lipopolysaccharide (LPS) for 24 hours. Cells were collected and stained with Annexin V-FITC and PI. n = 6 mice. Results for 1 representative experiment of 3 are shown. (TIF) [file pone.0090293.s005.tif]

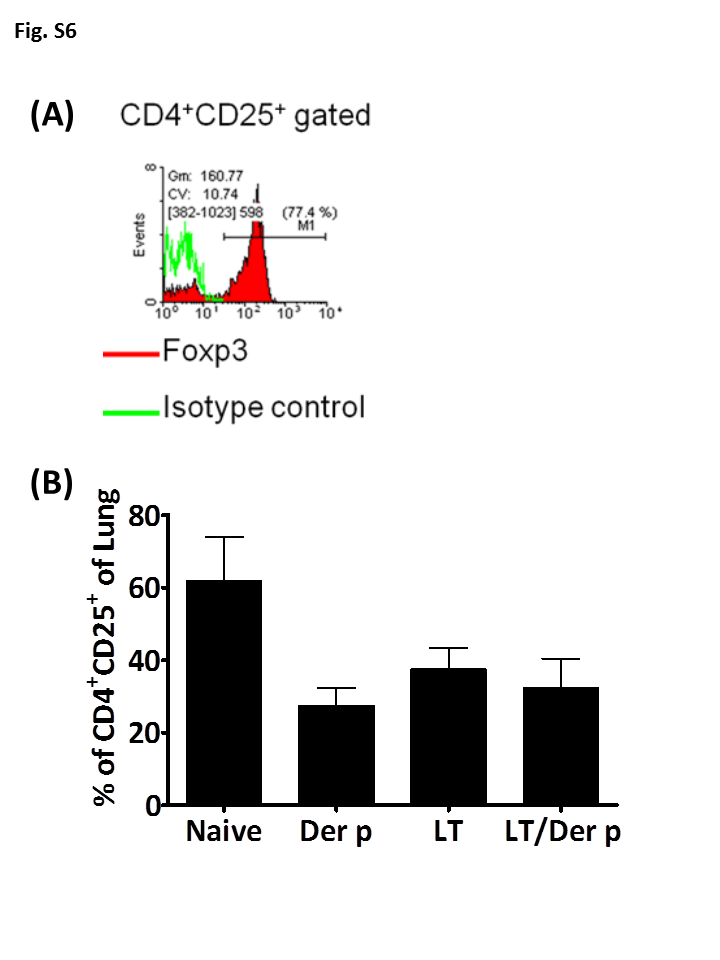

Supplement: Figure S6 — (A) Flow cytometry analysis of CD4+/CD25+ cells in collagenase-digested lungs. (B). Percentages of CD4+CD25+ cells in the lungs of naïve and treated allergic mice. (TIF) [file pone.0090293.s006.tif]
